# Computational Psychometrics in Communication and Implications in Decision Making

**DOI:** 10.1155/2015/985032

**Published:** 2015-08-03

**Authors:** Pietro Cipresso, Daniela Villani, Claudia Repetto, Lucia Bosone, Anna Balgera, Maurizio Mauri, Marco Villamira, Alessandro Antonietti, Giuseppe Riva

**Affiliations:** ^1^Applied Technology for Neuro-Psychology Lab, IRCCS Istituto Auxologico Italiano, Via Ariosto 13, 20145 Milan, Italy; ^2^Department of Psychology, Catholic University of the Sacred Heart, Largo Gemelli 1, 20123 Milan, Italy; ^3^Social Psychology Research Group (EA 4163), Université Lumière Lyon 2, 5 avenue Pierre Mendès-France, 69656 Bron, France; ^4^IULM University-Milan, Via Carlo Bo 8, 20143 Milan, Italy

## Abstract

Recent investigations emphasized the role of communication features on behavioral trust and reciprocity in economic decision making but no studies have been focused on the effect of communication on affective states in such a context. Thanks to advanced methods of computational psychometrics, in this study, affective states were deeply examined using simultaneous and synchronized recordings of gazes and psychophysiological signals in 28 female students during an investment game. Results showed that participants experienced different affective states according to the type of communication (personal versus impersonal). In particular, participants involved in personal communication felt more relaxed than participants involved in impersonal communication. Moreover, personal communication influenced reciprocity and participants' perceptions about trust and reciprocity. Findings were interpreted in the light of the Arousal/Valence Model and self-disclosure process.

## 1. Introduction

Recently attention has been focused on the study of the effects of different types of communication on behavioral trust and reciprocity. Typically, the trust game is one of the preferred contexts to study trust and reciprocity in decision-making situations [1, 2].

As far as the role of communication in decision making is concerned, it is possible to identify two different perspectives. On the one hand, Bicchieri and colleagues defined* communication effect* [3, 4] as the positive effect of face-to-face (FtF) communication on cooperation and, particularly, on prosocial behavior. Specifically, oral communication led to the* identification* (humanization) of other agents. When another subject is perceived as being similar to ourselves—even if the similarity is vague and generic—we have the tendency to be kinder and more generous than we would be if the other part were completely anonymous. Identification reduces what is called “social distance” and therefore may increase the scope for reputation effects, which in turn may yield more cooperation [5]. In particular, Bicchieri [3, 6] distinguished between “relevant” and “irrelevant” communication. The former refers to the strategic discussion of the game and promise-making, usually producing more trust and cooperation than “irrelevant” communication [7].

Within the game-irrelevant communication, Buchan and colleagues [8] introduced a distinction between “personal” (e.g., subjects introduced themselves and talked about birthdays) and “impersonal” (e.g., subjects answered questions from the world almanac). Authors found that personal, game-irrelevant communication has a powerful influence on trusting behavior and they explained this result by stating that “the mere act of communicating more about themselves on a personal topic prompted participants to be significantly more concerned with others” [8].

As far as we know, the effects of communication on decision have been studied only in terms of behavioral results, such as the amount of money sent by the proposer (trust) and the amount of money sent by the responder (reciprocity). No studies have been focused on what happens during the communication process between participants in terms of affective and cognitive states.

To investigate this process we used computational psychometrics that has several advantages. First, this approach allows extracting several critical pieces of information related to subjective processes, without asking directly individuals. Second, thanks to the availability of brand-new technologies and paradigms researchers can investigate communication without interfering with the communication flow. Third, the use of computational psychometrics allows overcoming limits related to the use of the classic psychometrics questionnaires, where individuals are asked to recall* a posteriori* the communication experience.

## 2. Psychophysiological Correlates of Communication

At our best knowledge no study investigated yet the relationship between affective states and communication type. On the other hand several studies investigated the relationship between affective states and psychophysiological correlates in individuals by using biosensors [9].

To classify participant's affective states during communication, we referred to the well-known Arousal/Valence Model [10, 11], based on two dimensions: physiological arousal (high versus low) and emotional valence (positive versus negative). Lang and colleagues [12] found close relations between specific psychophysiological measures (e.g., corrugator and zygomatic electromyographic activity) and the self-reported valence dimension of emotion and between other psychophysiological measures (i.e., electrodermal activity) and the self-reported arousal dimension.

This approach has been extensively used in psychophysiological research as an objective way to measure affective states during a mediated experience [13–23]. More recently an extensive research has been done to discern different emotions by means of cardiovascular measures [24] and this result hugely helps the analysis of affective states in terms of cardiovascular indexes patterns [25–27]. For example, in a recent study Causse and colleagues [28] examined pilots' cardiovascular and oculometric measurements related to decision making.

To identify the affective and cognitive states with regard to the type of communication, we followed the process tracing paradigm [29] and we used an integrated psychophysiological approach. This approach is based on two methods frequently used to study behaviors and emotions, such as psychophysiological correlates [30] and eye movements recording [31–34]. Thanks to this integrated psychophysiological approach we aimed to overcome two principal limits related to the use of self-reports and behavioral data for assessment. At first, we would potentially index important processes which underlie affective responsivity but are not accessible to consciousness [35]. Secondly, we were interested in assessing emotional responding during the communication without interfering with the process. In this sense, psychophysiological measures allow us to continuously measure the temporal course of affective responding. The more common alternative of obtaining self-reports immediately after the episode is completed does not assess the temporal course of affective responsivity and may not even be particularly accurate indices of individuals' integrated responsivity over the course of the episode [36, 37].

## 3. Goal and Hypotheses

The general goal of this study was to investigate the effects of personal and impersonal communication on participants' affective states during the communication phase and their implications for trust, reciprocity, and participants' perception about trust and reciprocity after the trust game. For this reason we used an investment game as a typical decision-making task and we considered only the affective states during the communication phase (i.e., the period in which participants discuss options before making the decision). This is the first study carried out by using double eye-tracker synchronized with double psychophysiological biosensors.

In particular, we formulate two hypotheses:

(H1) There are significant differences between participants' affective states depending on the topic of communication (personal versus impersonal); since no study investigated this specific issue we decided to explore this relationship by referring to the Lang Model.
(H2) There are significant differences between participants' behaviors in terms of trust and reciprocity depending on the topic of communication (personal versus impersonal). According to Buchan et al. [8], we expected higher level of trust and reciprocity in personal communication condition. Furthermore, we would like to investigate also the perceptions related to the choice after having made the decision and to do that we analyzed participants' indications through their answers to specific questions.

## 4. Materials and Methods

### 4.1. Ethics Statement

Ethical Committee of the Psychology Department of the Catholic University of Milan approved the study. All participants gave written informed consent to the experimental procedure according to the rules of the scientific review board.

### 4.2. Research Design

The experimental investigation relies on the investment game, introduced by Berg and colleague [38], which provides a nice environment for the observation of trusting behavior in the lab. The between-subject design was based on the communication content factor suggested by Buchan et al. [8] (personal communication, PC, versus impersonal communication, IC). In the PC condition participants were allowed to discuss matters regarding personal interests, identity, ideas, and so forth. In the IC condition participants were limited to discuss arguments not linked to personal issues, such as the weather, public transportation, and life in the city where they lived.

### 4.3. Participants

Thirty-two students (mean age = 23.21 yrs.; SD = 2.38; range = 21–33) attending the Faculty of Psychology at the Catholic University of Sacred Heart of Milan, Italy, took part in the experiment. All the participants were students in psychology or communication, but without previous knowledge of decision science and in particular with no knowledge of the investment game used in the experiment. Social, economic, cultural, and historical background were all similar since all the participants were students in the same university and with similar background. To avoid differences in money perception the level of incomes was similar (student workers have been excluded). They were first met by one of the researchers during academic courses and then contacted via mail and/or telephone to schedule a meeting at the psychophysiology laboratory. The topics associated with the experiment were not mentioned during academic courses.

We selected only women in order to reduce the effects due to confounding variables associated with gender, such as sexual attraction, and control a priori effect of identification [39].

The participants were informed that the reimbursement for their attendance was related to their choices in the game.

Four of the participants failed to complete the experimental session due to personal or technical problems and were excluded from statistical analyses. The final sample was composed of 28 students, who were randomly coupled and assigned to one of the two experimental conditions (PC versus IC).

### 4.4. Procedure

The experimenters attached the following biosensors: a respiration belt (RSP), positioned on the chest; a facial EMG on corrugator supercilii muscle (i.e., EMG-CS), placed on the forehead after the researcher cleaned the skin with abrasive paste; two EEG sensors located on the frontal lobes, on the right and on the left, respectively, attached with conductive paste to improve signal detection (standard position on FP1 and FP2 of the EEG 10–20 international electrode system), with the reference electrodes attached to the respective ear lobes after they had been cleaned with abrasive paste by the experimenter; two SC adhesive patches applied on the nondominant palm; a BVP sensor placed on top of the index finger of the nondominant hand.

Finally, participants' eye movements were calibrated by asking participants to follow a small point on the screen. The EEG and the physiological signals of BVP, GSR, respiration, and EMG-CS were acquired using a ProComp Infiniti device from Thought Technology, including Biograph Infiniti 5.0.2 software to record all signals. Every channel was synchronously acquired at 2048 Hz and exported at a 256 Hz sampling rate (every 3.90625 milliseconds).

In order to analyze deeper the affective states related to different types of communication content during the communication phase in FtF condition, we used video-conferencing technologies. According to Brosig et al. [39], video-conferencing is useful for employing the favorable features of FtF communication as a “real” conference and it produces cooperation rates very close to FtF communication [40].

Both members of each pair were separately welcomed in different rooms by two experienced experimenters, who assisted them for the duration of the laboratory session. The experimenters were instructed to maintain a neutral voice tone and a neutral behavior while the subjects were being exposed to experimental stimuli. Participants did not know each other and could not talk to each other before the beginning of the experiment. First of all they were informed about the general purpose of the research (summarized in the sentence: “The goal of the experiment is to analyze the different ways people take a decision”) and received all the instructions about the nature of the investment game (including the switch of the roles). Then, they signed the written informed consent and were prepared in order to collect the psychophysiological data. When a participant indicated feeling comfortable, the researcher asked her to try to remain still during all the experimental conditions in order to avoid artifacts in signal acquisitions as a result of movements. A baseline measure of the psychophysiological parameters was obtained with a 5-min registration in a steady state [27].

At this point the experimental task started. The two participants were allowed to get to know each other, meeting their partner in the virtual context of Google video chat. The communication lasted 3 min. They were asked to converse about specific arguments among authorized topics, according to the experimental condition to which they belonged (PC versus IC).

After this step, the decision phase started and participants knew that the game was played with real monetary payoffs and the winnings were converted in a proportioned way.

The proposer was asked to choose how much money she would give to the partner from an initial amount of 2000 Euros by choosing one of the following alternatives: 100, 500, 1000, 1500, and 2000 Euros. According to the investment game procedure, the responder was then asked to choose how much of the triple of the amount received she wanted to give back to the proposer, selecting one of the following options: nothing, 1/4, 1/2, or 3/4 of the final amount and all the final amount.

After the decision, the video chat was closed and participants completed a brief questionnaire about the choice they had made and related personal motivations and the partner's choice and related motivations.

The second phase of the experiment started with another 3-min video-chat communication, when the game was repeated, by inverting the roles.

### 4.5. Signal Acquisition and Data Analysis

The experiment was carried out in two labs, each one equipped with two portable PCs, one for delivering the stimuli and acquiring eye-tracker data and the other for psychophysiological signal recording.

The physiological signals were acquired using a ProComp Infiniti device from Thought Technology, including Biograph Infiniti 5.0.2 software to record and export all raw signals. Every channel was synchronously acquired at 2048 Hz and exported at least a 256 Hz sampling rate (every 3.90625 milliseconds) or higher where needed.

The pupillometry data were acquired using two Tobii x-series, including Tobii Studio software to record all raw signals, then exported, and resampled at 60 Hz.

In our study, by using the eye-tracker data extraction, we obtained for each participant a matrix of gaze and pupil data corresponding to stimuli presentation (such as the webpage for Google video chat); in particular, we collected 50 rows for each second (sampling to 50 Hz), thereby making it possible to establish the exact period previously indicated.

Then, the synchronization of the psychophysiological signals allowed us to identify the communication period. In this case, we used several algorithms to synchronize eye-tracker systems with a ProComp Infiniti device from Thought Technology by using the TT-AV Sync sensor, which was configured through a physical channel on ProComp capturing the light (e.g., by identifying black and white) thanks to a photodiode actually applied on the screen [41]. Moreover, based on gazes and pupil signals acquired, it has been possible to identify eye-blinks, which enabled us to align the matrix containing the eye-blink data from gazes and pupil signals, with the matrix containing the psychophysiological signals. Thanks to this procedure, we correctly identified the communication period with an error of ±0.01 seconds.

All collected biosignals were analyzed using Matlab 7.0 (The Mathworks, Natick, MA, USA) for the signal processing and computation of psychophysiological measures.

Data were analyzed with the aid of the statistical software SPSS, version 18 (Statistical Package for the Social Sciences—SPSS for Windows, Chicago, IL, USA).

### 4.6. Psychophysiology and Affective States

According to the classic valence-arousal model [10, 11] described in Introduction, we considered the two dimensions of physiological arousal and emotional valence for identifying affective states in participants during the experimental session.

Physiological arousal can be measured by using electroencephalogram (EEG), galvanic skin response (GSR), cardiovascular activity (ECG or BVP), and respiration signal (RSP); emotional valence can be measured by using EEG, self-reports, facial expression identification, eye-blink startle, and facial EMG corrugator and/or zygomatic. According to Blumenthal and colleagues [42], facial EMG-CS (corrugator) can be considered the best measure for emotion valence.

Frontal EEG activation asymmetry has been generally used, giving evidences that greater left frontal activity seems to be higher related to positive emotional valence, whereas greater right frontal activity seems to be more involved in negative emotional valence [43]. Alpha index seems to be the most adaptation to study the frontal EEG activation asymmetry [43]. Thus, Alpha Asymmetry index can be calculated in many different ways to take into account the hemispheric prevalence and to correct the sign accordingly. In calculating this index it is crucial to consider that higher cortical activation is revealed by lower Alpha waves, and thus this needs to be taken into account in the computation and formula derivation.

**Figure 1:**
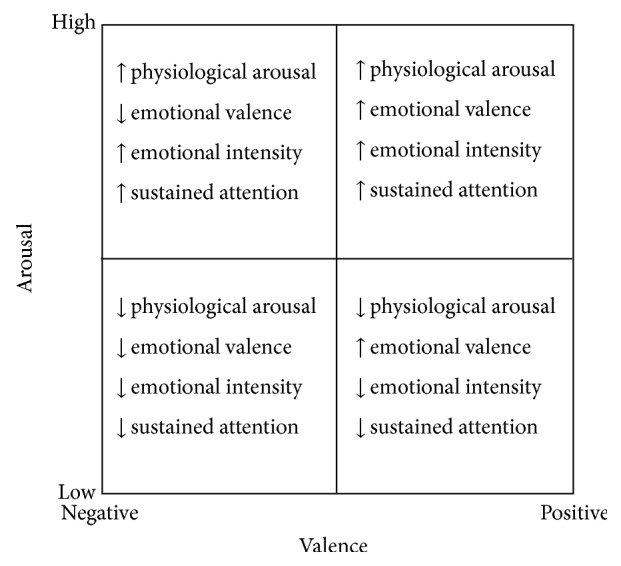
The affective space, defined by the dimensions valence and arousal, based on Lang [10] and the physiological activation in central or peripheral nervous system.

Furthermore, we used pupillometry to investigate emotional responses. Because pupil dilation indicates a higher cognitive load or emotional processing [25, 27], studies have suggested that combining pupillometry and the use of functional magnetic resonance imaging (fMRI) to produce a pupil dilation index can provide summative information on cognitive and affective processes [44].  Figure 1 provides the relationships between psychological affective states and physiological “activation” increasing (↑) and decreasing (↓).

As recently showed by Mojzisch and colleagues [45], the self-involvement during social interactions is specifically related to attention allocation. For this reason we are interested also in investigating the sustained attention as another dimension of analysis. Slow Alpha EEG bands (following Slow Alpha) from EEG have been demonstrated to be a valid measure of sustained attention [46, 47].

### 4.7. Signal Processing

Cardiovascular and respiratory activities were monitored to evaluate both voluntary and autonomic effect of respiration on heart rate, analyzing IBI (Interbeat-Interval extracted from Blood Volume Pulse sensor, recognized as a measure equivalent to R-R interval extracted from electrocardiogram), respiration (from chest strip sensor), and their interaction. Following the guidelines of task force of the European Society of Cardiology and the North American Society of Pacing and Electrophysiology, typical Heart Rate Variability (HRV) temporal and spectral method indexes were used to evaluate the autonomic nervous system response [48–52]. Temporal indexes were computed from IBI as indicated in the guideline as heart rate (HR), number of pairs of successive NNs (derived from IBIs) that differ by more than 50 ms (millisecond), namely, NN50, and Root Mean Square Standard Deviation (RMSSD). Spectral analysis was performed using Fourier spectral methods with custom software. The rhythms have been classified as very low frequency (VLF, < 0.04 Hz), low frequency (LF, from 0.04 to 0.15 Hz), and high frequency (HF, from 0.15 to 0.5 Hz) oscillations. This procedure enabled us to calculate the LF/HF ratio, also known as the sympathovagal balance index. More, we were able to check if variations in ECG signal were due to variation in respiration affected by talking. An accurate checking of LF, respiration rate, and a simple analysis of respiratory sinus arrhythmia allowed us to estimate no effects of the talking on ECG signals. All artifacts in ECG signals have been corrected, according to the guidelines [52] and Clifford and colleagues [53].

SC and skin resistance recorded through GSR sensors are units of electrodermal activity, which are expressed in either conductance (microsiemens) or resistance (microohms). SC reflects a fairly slow physiological process and can be sampled at 32 Hz without distortion. The signal is expressed in microohms. We considered mean and standard deviation of the sampled signal.

The raw electromyography is a collection of positive and negative electrical signals. Their frequency and amplitude gave us information on the contraction or rest state of the muscle. Amplitude was measured in *μ*V (microvolts). As the participant contracts the muscle, the number and amplitude of the lines increase; as the muscle relaxes, it decreases. We considered the Root Mean Square (RMS) for rectifying the raw signal of the corrugator supercilii muscle and converting it to an amplitude envelope, following EMG-CS. We were not interested in frequency related to this muscle fatigue.

EEG signals need to be extensively worked to remove ocular artifacts and blinks using automatic algorithm and subsequent visual inspection. Then the corrected matrixes have been computed to calculate means of the Slow Alpha bands (at 7–10 Hz) for the FP1 and FP2 EEG channel, through spectral analyses [54–56]. Eye-tracker usefulness in this study was also critical for synchronizing the psychophysiological signals within the communication phase (based on a photodiode applied on the eye-tracker monitor and connected as a channel to the psychophysiology control unit).  Table 1 offers a clear indication of the variables measured as described above.

### 4.8. Questions Asked after the Decision

After the decision, the video chat was closed and participants completed a brief questionnaire. In particular, proposers were asked about their choice and the personal motivations for it through two questions:

- How do you consider the amount of money you gave to the responder? (“Big” or “Small”);
- Why did you give to your partner this amount of money? (“Because she seemed trustworthy,” “Because she did not seem trustworthy,” “Because she seemed generous,” or “Because she did not seem generous”).

Responders too were asked about their choice and the personal motivations for it through two questions:

- How do you consider the amount of money you gave back to the proposer? (“Fair,” “Partially fair,” or “Unfair”);
- Why did you give to your partner this amount of money? (“Because it was the right choice,” “Because she would have done the same,” “Because the gain had to be shared equally between me and her,” “Because at the beginning it was her money,” and “Because thanks to me the money have been increased”).

## 5. Results

The dependent variables considered were defined as follows:

- Psychophysiological measures (extracted following the above indication; see 2.7): heart rate, EMG corrugator, skin conductance, EEG Alpha Asymmetry, EEG Slow Alpha waves, EEG Beta waves, LF/HF, pupil dilation, NN50, and RMSSD.
- Trust: the amount of money sent by the proposer to the responder.
- Reciprocity: the amount of money returned by the responder to the proposer.
- Perceived trust: the individual's evaluation of fairness of personal decision about the amount of money sent to the responder.
- Perceived reciprocity: the individual's evaluation of fairness of personal decision about the amount of money sent to the proposer.

Analyses were carried out in order to test the hypotheses. For this reason we analyzed differences between participants' affective states depending on the topic of communication (H1) and differences between participants' trust and reciprocity and their perceptions about trust and reciprocity depending on the topic of communication (H2).

### 5.1. Comparison between Participants' Affective States Depending on the Topic of Communication (H1)

We analyzed the effect played by the independent variable “role” (proposer versus responder) on all dependent variables: since it resulted not statistically significant in both main and interaction effects, it was not further considered in the subsequent analyses.

The impact of communication type on psychophysiological measures of affective states was evaluated by running a series of independent samples* t*-tests in a between-factor design (PC versus IC) to compare the two groups of participants.* t*-test correction has been done whether or not dependent variable variance between groups was homogeneous, based on Levene's test.

The descriptive statistics summarized in Table 2 and the corrected* t*-tests in Table 3 showed that participants in the PC condition had lower HR and SC, indicating a lower physiological arousal than participants in the IC condition. On the other hand lower EMG corrugator and lower Alpha Asymmetry indexes for participants in the PC condition indicated a positive emotional valence but a negative emotional valence in participants in the IC condition. Moreover, results showed a lower sympathovagal balance index (LF/HF ratio) and higher RMSSD and NN50. This last triplet of indexes, with these trends, still demonstrated a state characterized by positive valence and low arousal in the PC condition and a state characterized by negative valence and high arousal in IC condition. Pupil dilation showed statistical significant differences between conditions, indicating that emotional intensity was higher in IC. Negative emotions had a stronger effect on states characterized by negative valence and high arousal than positive emotions on states characterized by positive valence and low arousal did.

Finally, Slow Alphas were lower in participants in the PC condition, highlighting their higher sustained attention than in IC.

A power analysis was used to anticipate the likelihood that our study yielded significant effects. The goal of our post hoc power analysis was to compute achieved power, given significance level, effect size, and sample size. The analysis showed a good power (above 80% per each measure). These results also demonstrated that our sample is at the fair size.

### 5.2. Comparison between Participants' Trust and Reciprocity Depending on the Topic of Communication and Their Perceptions about Trust and Reciprocity after the Choice (H2)

The impact of communication type on trust and reciprocity was evaluated by running a series of independent* t*-tests (see Tables 4 and 5 for frequencies). No differences were found between the amount of money sent from the proposer to the responder in the PC and IC conditions (*t* = 0.577, df = 26, and *P* = .569). As far as reciprocity was concerned, results suggested that responders returned a higher amount of money to the proposers in the PC condition (*t* = 2.096, df = 26, and *P* = .046). Nobody gave back zero or the total final amount.

We investigated also the participants' perceptions about their choices by using Kendall tau-c and we compared them depending on the communication condition.

Analyses showed that differences exist between participants' perceptions about trust depending on the topic of communication. In particular, the motivations related to the proposer's play were different between PC and IC (tau_*c*_ = −0.384, df = 26, and *P* = .032): the proposers in PC condition considered the responders more trustworthy than in IC condition.

As a consequence, responders evaluated the personal decision about the amount of money returned to the proposers as fairer in the PC condition (tau_*c*_ = 0.459, df = 26, and *P* = .020).

## 6. Discussion and Conclusions

According to Bicchieri [3, 6], the content of communication plays a key role during the interaction in decision making. In particular, according to Buchan et al. [8], we decided to concentrate our study on the “irrelevant communication” by differentiating between “personal” irrelevant communication condition and “impersonal” irrelevant communication condition.

The general aim of this study was to investigate the effects of personal and impersonal communication on participants' affective states during the communication phase preceding a decision and on their perception about trust and reciprocity after the investment game. A cutting-edge innovation was represented by the computational psychometrics approach, using double eye-tracker synchronized with double psychophysiological biosensors.

As far as the first hypothesis is concerned, we found differences between participants' affective states depending on the topic of communication. We also verified that psychophysiological correlates of affective states experienced during the communication phase remained stable and were confirmed during the decision phase.

In particular, according to the Arousal/Valence Model [10, 11], psychophysiological measures showed two different profiles. Participants in the PC condition were more relaxed than participants in the IC condition and positive emotional valence, low arousal, and high attentional resources characterized their affective states. On the other hand, participants in the IC condition were more activated than participants in the other condition and low emotional valence, high arousal, and low attentional resources characterized their affective states.

Probably participants who were allowed to speak about personal interests, identity, and ideas felt low activated and experienced positive states. It is possible to place these individuals in the affective dimension of positive valence and low arousal of the Lang Model (see Figure 1). On the other hand, participants who were limited to discussing arguments not linked to personal issues felt highly activated and uncomfortable. It is possible to place these persons in the affective dimension of negative valence and high arousal of the Lang Model (see Figure 1).

This sense of being comfortable could be explained by the fact that in personal communication condition participants were involved in a self-disclosure process. Self-disclosure has been defined as any message about the self that an individual communicates to another [57, 58]. According to traditional interpersonal theories, self-disclosure is a type of communication through which individuals make themselves known to other people and when others reciprocate by sharing revealing information it leads to intimacy and relational development [59]. Furthermore, according to the uncertainty reduction theory, individuals will not only seek information to reduce uncertainty but also reciprocate with similar amounts of information and at the same level of intimacy [60]. The self-disclosure process could have got participants in personal communication condition closer to each other and more comfortable.

Psychophysiological correlates supported the self-disclosure process also regarding sustained attention. Indeed, participants in the PC condition showed high sustained attention even if their other states fitted with the relax dimension. Participants belonging to the impersonal communication showed low sustained attention even if their other states fitted with the negative valence and high arousal affective state. Probably the personal content of communication led participant to feel engaged in friendly and attentive interaction with the partner [61].

According to our results, the second hypothesis was partially supported. The feeling of being relaxed and more comfortable during personal communication was not associated with proposer's trust, even if proposers in PC considered the responders as trustworthy. However, the positive emotional valence, low arousal, and high attentional resources featured by participants in PC condition were associated with a higher amount of money returned to the proposer and an evaluation about the amount of money returned to the proposers as fairer than in the IC condition.

To summarize, we found differences between psychophysiological correlates of communication conditions related to the affective and cognitive states. Furthermore, we found a significant difference related to the choices and participants' perceived trust and reciprocity according to the content of communication.

Many different issues are still open and deserve further investigation. First of all, although we selected only women in order to reduce the effects due to confounding variables associated with gender, future researches should include men in order to verify whether gender differences exist. Second, since the investigation of the relationships between the affective states during personal versus impersonal communication and the behavioral responses was out of the purpose of the present research, we did not try to pinpoint causal links between the two. However, the next step would be to build predictive models able to identify the affective states that lead to differences in trusting behavior. Third, we opted for a single-shot trust game instead of iterative one. This choice fitted our experimental goals and a future challenge would be to repeat the experimental setting in multiple rounds of the trust games in order to investigate the effects of repeated communications on trust: does the deepening of the self-disclosure (in PC) versus the repeated talk about generic topics (in IC) change the trust over time?

The present results suggest that a personal conversation is more oriented to positive affective states and to positive perceptions related to the partner of communication. Implications could be relevant for many purposes, since many communication processes made in both FtF and online settings can precede decision making. Vivid examples are related to the physician-patient communication [62, 63], both FtF and online, where the physician can be rated as more empathic thanks to the open communication used [64] or also the insurance and financial fields, where online decision making is rapidly becoming more relevant than ever. In both cases, a personal communication could help patients and clients, such as the responders of real-life decisional situations, to feel more comfortable and this affective state could lead them to be more favorably disposed during the decisional process.

Thanks to the use of computational psychometrics we have been able to investigate communication processes and their effects on decision making. In particular, the use of double systems synchronized to record the related psychophysiological correlates and eye movements has shown great potential.

The extended use of new computational psychometrics paradigms represents a promising approach and future researches aiming to investigate communication processes are encouraged to follow these advances methods.

## Figures and Tables

**Table 1:** Description of variables and their measures (↑ is for increase; ↓ is for decrease).

Variable	Measures
↑physiological arousal	**↑**heart rate/**↑**skin conductance/**↑**EEG Beta waves
↑emotional valence	**↓**EMG corrugator/**↓**EEG Alpha Asymmetry
↑emotional intensity	**↑**pupil dilation
↑sustained attention	**↓**EEG Slow Alpha waves
↑negative affect and activation	**↑**LF/HF/**↓**NN50/**↓**RMSSD

**Table 2:** Descriptive statistics of physiological measures for the two conversation groups (PC versus IC).

Measure	Condition	*N*	Mean	Std. deviation	Std. error
Heart rate^*∗*^ (*physiological arousal*)	Personal	14	1.169537	0.282227	0.075428
	Impersonal	14	1.492253	0.514155	0.137414

EMG corrugator^*∗*^ (*emotional valence*)	Personal	14	1.308825	0.296176	0.079156
	Impersonal	14	1.704306	0.590298	0.157764

Skin conductance^*∗*^ (*physiological arousal*)	Personal	14	1.310103	0.305998	0.081782
	Impersonal	14	1.744871	0.644443	0.172235

EEG Alpha Asymmetry^*∗*^ (*emotional valence*)	Personal	14	1.619759	0.331382	0.088566
	Impersonal	14	2.034656	0.613816	0.164049

EEG Slow Alpha waves^*∗*^ (*sustained attention*)	Personal	14	1.655140	0.281301	0.075181
	Impersonal	14	1.996572	0.529739	0.141579

EEG Beta waves^*∗*^ (*physiological arousal*)	Personal	14	1.270383	0.331340	0.088554
	Impersonal	14	1.656981	0.619189	0.165485

LF/HF (*Heart Rate Variability*)	Personal	14	1.230078	0.272284	0.072771
	Impersonal	14	1.474582	0.429780	0.114864

Pupil dilation^*∗*^ (*emotional intensity*)	Personal	14	.4252	.09369	.02504
	Impersonal	14	.5309	.16404	.04384

NN50 (*Heart Rate Variability*)	Personal	14	0.699334	0.132247	0.035344
	Impersonal	14	0.603127	0.173188	0.046286

RMSSD^*∗*^ (*Heart Rate Variability*)	Personal	14	0.674883	0.306916	0.082027
	Impersonal	14	0.372111	0.422183	0.112833

^*∗*^ *P* ≤ .055 according to corrected *t*-tests showed in Table 3.

**Table 3:** Physiological measures: between condition effects (corrected *t*-tests).

Communication type (personal versus impersonal communication)				
	Mean difference	*t*_26_	St. Err.	Sig.
Heart rate	−0.3227	−2.059	0.1567	.053
EMG corrugator	−0.3955	−2.241	0.1765	.037
Skin conductance	−0.4348	−2.280	0.1907	.035
EEG Alpha Asymmetry	−0.4149	−2.225	0.1864	.038
EEG Slow Alpha waves	−0.3414	−2.130	0.1603	.046
EEG Beta waves	−0.3866	−2.060	0.1877	.053
LF/HF	−0.2445	−1.798	0.1360	.084
Pupil dilation	−0.2031	−2.029	0.1001	.046
NN50	0.0962	−1.652	0.0582	.111
RMSSD	0.3028	−2.170	0.1395	.039

**Table 4:** Money sent by the proposer to the responder.

Valid	Personal communication		Impersonal communication	
	Frequency	Percent	Frequency	Percent
100	0	0	2	14.3
500	1	7.1	3	21.4
1000	7	50	3	21.4
1500	4	28.6	2	14.3
2000	2	14.3	4	28.6
Total	14	100	14	100

**Table 5:** Money returned by the responder to the proposer.

Valid	Personal communication		Impersonal communication	
	Frequency	Percent	Frequency	Percent
1/4	1	7.1	3	21.4
1/2	6	42.9	9	64.3
3/4	7	50	2	14.3
Total	14	100	14	100
